# Foundations and Advancements in Hemodynamic Monitoring: Part I-Elements of Hemodynamics

**DOI:** 10.4274/TJAR.2025.251925

**Published:** 2025-05-30

**Authors:** Zeliha Aslı Demir, Emre Sertaç Bingül, Burhan Dost, Gamze Talih, Aslıhan Aykut, Muhammed Enes Aydın, Başak Akça, Ümit Karadeniz

**Affiliations:** 1 Ankara Bilkent City Hospital, Clinic of Anaesthesiology and Reanimation Clinic, Ankara, Türkiye; 2 İstanbul University, İstanbul Faculty of Medicine, Department of Anaesthesiology and Intensive Care, İstanbul, Türkiye; 3 Ondokuz Mayıs University Faculty of Medicine, Department of Anaesthesiology and Reanimation, Samsun, Türkiye; 4 Erciyes University Faculty of Medicine, Department of Anaesthesiology and Reanimation, Kayseri, Türkiye; 5 Atatürk University Faculty of Medicine, Department of Anaesthesiology and Reanimation, Erzurum, Türkiye; 6 Hacettepe University Faculty of Medicine, Department of Anaesthesiology and Reanimation, Ankara, Türkiye

**Keywords:** Anaesthesia monitoring, hemodynamics, intensive care, patient outcomes, perioperative care

## Abstract

Standard monitoring guidelines by the American Society of Anesthesiologists and European Society of Anaesthesiology and Intensive Care have not been updated for over a decade, despite rapid advancements in monitoring technology and the growing complexity of surgical patients. Traditional parameters such as blood pressure and pulse oximetry often fail to detect critical intraoperative conditions, emphasizing the need for comprehensive hemodynamic assessment. This review, the first of a two-part series, explores the fundamental elements of hemodynamics, including cardiac output, stroke volume, blood pressure, and oxygen delivery, with a focus on their physiological basis, clinical significance, and perioperative applications. This article provides a detailed foundation for understanding hemodynamic monitoring, setting the stage for the second article, which addresses advanced monitoring tools and their applications in contemporary anaesthesia practice.

**Main Points**

• Fundamental hemodynamic parameters, including cardiac output, stroke volume, blood pressure, and oxygen delivery, are essential for maintaining adequate tissue perfusion and ensuring timely intervention in perioperative care.

• Cardiac output, as a product of heart rate and stroke volume, provides a key indicator of macrohemodynamic stability, while stroke volume is modulated by preload, afterload, and myocardial contractility.

• Blood pressure monitoring alone may not accurately reflect tissue perfusion; understanding its relationship with systemic vascular resistance and flow dynamics is critical for clinical decision-making.

• Oxygen delivery depends on cardiac output and arterial oxygen content, emphasizing the importance of markers such as ScvO_2_, lactate, and the Pv-aCO_2_ difference in detecting hypoperfusion and guiding resuscitation.

• Emerging and future technologies in hemodynamic monitoring aim to integrate global and microcirculatory parameters, improving diagnostic precision and outcomes in anaesthesia and critical care.

## Introduction

The elements of standard monitoring defined by the American Society of Anesthesiologists were last updated in 2011, and the European Society of Anaesthesiology and Intensive Care also published a similar guideline in 2012.^1^ While efforts have been made in recent years to establish minimum monitoring criteria, the rapid advancement of technology, artificial intelligence, and monitoring algorithms some of which are now accessible via mobile phones has begun to make an impact.^2^ With the increase in life expectancy, the likelihood of encountering patients with many comorbidities undergoing major surgeries has risen significantly. For such patients, standard intraoperative monitoring may not simultaneously detect critical conditions such as hemorrhage, anaphylaxis, vasodilation, hypoperfusion of vital organs, ischemia, neurodepression, respiratory and cardiac depression, blood gas imbalances, fluid and electrolyte disturbances, acute heart failure, and arrhythmias. Timely intervention is crucial for saving the lives of critically ill patients, and this is only achievable through the prompt detection of complications. Normal readings of blood pressure (BP) and pulse oximetry do not necessarily indicate stability in critically ill patients; hence, close monitoring of physiological parameters is vital. In contemporary anaesthesia practices, monitoring of both macrohemodynamic and microhemodynamic parameters is expected to become increasingly prevalent to ensure adequate tissue perfusion (Table 1).

In this first article, Part I, we describe and discuss the fundamental elements of hemodynamics, focusing on their clinical importance and applications in the perioperative settings. This includes a detailed exploration of macrohemodynamic and microhemodynamic parameters essential for ensuring adequate tissue perfusion and timely intervention. In the companion article, Part II, we delve into advanced hemodynamic monitoring parameters and tools, highlighting their roles in addressing the limitations of standard monitoring and optimizing patient outcomes. Together, these two articles aim to provide a comprehensive understanding of hemodynamic monitoring for contemporary anaesthesia practices.

### Key Parameters and Physiological Concepts

### Cardiac output

**Figure 1:**
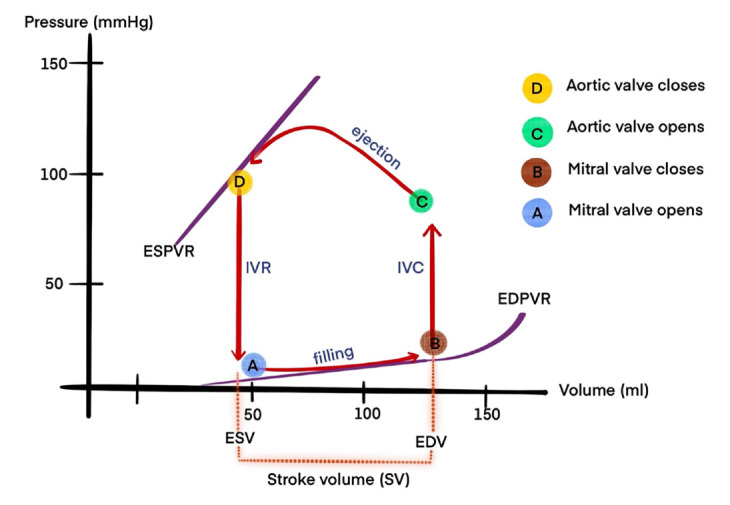
Left ventricle pressure volume loop IVR, isovolumic ventricular relaxation; ESPVR, end-systolic pressure-volume relationship; IVC, isovolumic contraction time; ESV, end-systolic volume; EDV, end-diastolic volume

Cardiac output (CO) is defined as the volume of blood ejected by the heart per minute, and is the product of stroke volume (SV) and heart rate (HR), expressed as CO=SVxHR (liters/minute). The amount of blood ejected during a single cycle is called SV, which averages around 70 mL in a resting adult. Atrial systole contributes to this by providing 15-25% of left ventricular diastolic filling without increasing the mean left atrial pressure. This contribution varies depending on the PR interval, atrial inotropic state, atrial preload and afterload, autonomic status, and HR. At the end of the diastole, the volume of blood in the ventricle is referred to as the end-diastolic volume (EDV), which is typically around 120 mL. The ventricles do not empty completely with each beat; only 60-80% of the blood is ejected (ejection fraction). Under resting conditions, physiological CO equates to approximately 4,900 (70 beats per minute ×70 mL), mL/min. Since patients of different sizes have varying COs, the cardiac index (CI) is often preferred for meaningful comparisons. CI is calculated as CO divided by body surface area (CI=CO/body surface area) (liters/min/m²).^3^ Pressure-volume changes in the left ventricle during a cardiac cycle are depicted in Figure 1.

The resting HR is approximately 70 beats per minute in a person with normal physiology, and therefore each cardiac cycle lasts about 0.85 seconds. The approximate duration of systole is 0.3 seconds while diastole lasts 0.55 seconds. An increase in HR shortens diastole, reducing the time available for ventricular filling, which consequently leads to a decrease in SV. Perfusion of the ventricular muscles predominantly occurs during diastole. As a result of shortening diastole, the duration of coronary blood flow is shortened, perfusion is consequently impaired, and the workload increases. This is the time when the heart is working at maximum capacity, resulting in a high oxygen demand and an increased risk of myocardial ischemia.

### Stroke volume

### Preload

The more a muscle fiber is stretched before being stimulated to contract, the greater the force of contraction becomes. However, this characteristic is limited by the internal molecular structure of the muscle, such that further stretching beyond a critical (optimal) point reduces the force of contraction. This feature is known as the Frank-Starling mechanism.^4^ During diastole, the blood returning to the heart stretches the ventricular muscle fibers. As the volume in the ventricle increases, the contraction becomes stronger. Thus, preload is directly related to EDV (or end-diastolic pressure as they change together), and if myocardial contractility and afterload are kept constant, increasing preload raises the EDV and consequently the SV. However, beyond a certain point, contractility begins to decline in overwhelmed muscle fibers.

In clinical practice, measuring EDV is challenging; therefore, estimations are made by measuring pressures. Central venous pressure (CVP) provides a reflection of right ventricular EDV, while pulmonary artery occlusion pressure reflects left ventricular EDV, although these values may not provide entirely accurate information.

### Contractility

Contractility is the heart’s ability to generate external work independently of preload and afterload. At the molecular level, an increase in inotropic state means stronger contractions due to increased calcium influx or myofilament calcium sensitivity, which results in higher peak pressure. Inotropy refers to the strength of the heart’s contraction, chronotropy refers to the HR, dromotropy describes the conduction speed of electrical impulses within the heart, and lusitropy represents the heart’s ability to relax. Positive lusitropic effects allow the heart to relax faster during diastole, enabling the ventricles to fill with more blood, thus enhancing CO. Negative lusitropic effects can lead to diastolic dysfunction.

### Afterload

**Figure 2:**
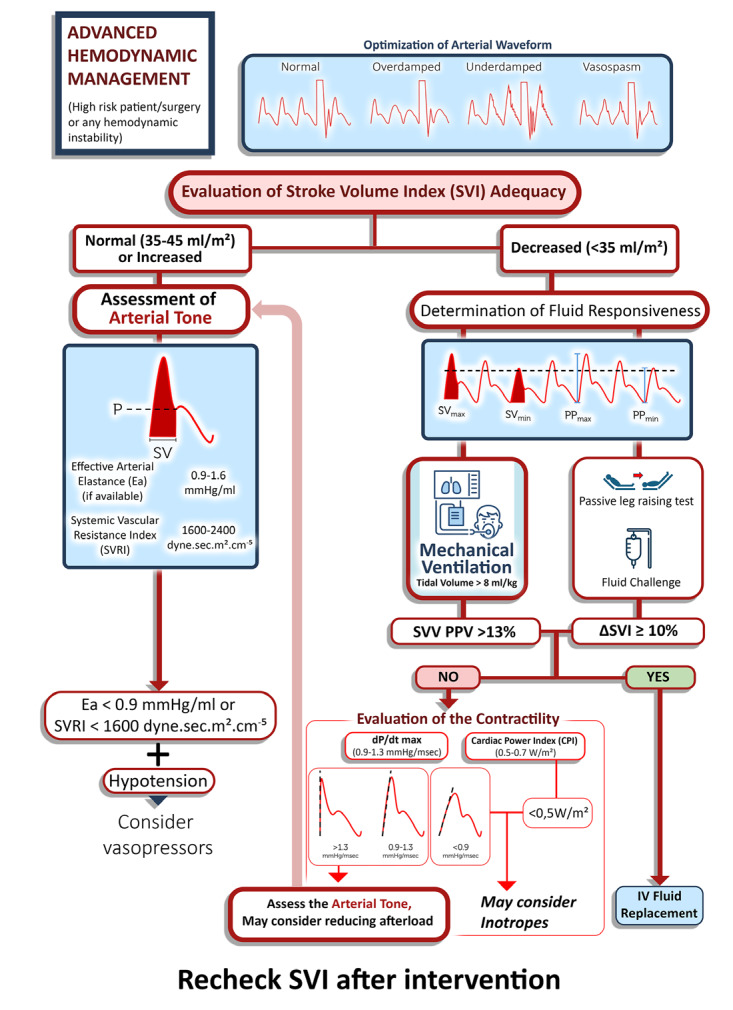
Algorithm for advanced hemodynamic management. This algorithm was designed as a simplified guide for the application of hemodynamic parameters. In clinical practice, patient-specific factors and varying clinical scenarios should be carefully considered when interpreting and utilizing an algorithm. Continuous reassessment and integration of additional physiological and diagnostic data are essential for optimizing hemodynamic management

At the end of diastole, when ventricular muscle begins to contract, the goal is to overcome the “tension” in the ventricular wall and the “resistance” opposing the ejection of blood from the ventricle. In fact, the arterial system is not a simple conduit but a complex tree that represents several physical features such as compliance, resistance, and impedance. Given this complexity, theoretical calculation of afterload is utterly difficult. When ventricular wall tension is neglected, left ventricular afterload corresponds to the resistance posed by systemic circulation, termed systemic vascular resistance (SVR). Conversely, right ventricular afterload pertains to the resistance encountered in pulmonary circulation, known as pulmonary vascular resistance. Afterload is predominantly influenced by vascular tone, or the degree of vasoconstriction (or dilation) within the arteries and arterioles. Consequently, an increase in SVR results in reduced blood ejection for a given preload and contractility, leading to more blood remaining in the ventricle at the end of systole and a subsequent decrease in SV.^5^ By recalling these fundamental concepts, one can gain practical experience through the application of our algorithm. The proposed algorithm is designed to provide clear guidance for hemodynamic monitoring, especially for novices (Figure 2).

### Right ventricular function

All the principles and discussions mentioned so far are also applicable to the right ventricle. Essentially, the myocytes of the right and left ventricles are the same, with only a few minor differences. The most significant difference between the two ventricles is in the geometry of the cavity, according to Laplace’s law, and the resulting pressure differences (the pressure in the right ventricle and pulmonary circulation is lower compared to the left). Because the right ventricle wall is thin, its shape changes quickly under varying conditions and is not adapted to contraction against high pressures. Therefore, in the presence of pulmonary hypertension, right heart failure appears more quickly and distinctly due to the excessive afterload.

### Blood pressure

In daily practice, BP monitoring is essentially provided, yet CO is rarely assessed. It is important to understand the relationship between these two, because pressure is not equivalent to flow. Patients can have a “normal” BP with much lower flow, or normal flow with significantly different BP. BP results from the heart pumping blood into the relatively small-capacity arterial system, which constitutes a significant resistance and contains only about 15% of the circulating volume. Resistance can be expressed in terms of pressure drop per unit of flow (according to Ohm’s law, V=IR); circulatory resistance is defined as BP/CO, so BP=CO×SVR.

According to the equation, in a patient with very low CO, the body can maintain BP up to a point by increasing SVR. All organs need a pressure difference between arteries and veins to ensure sufficient flow through the large capillary beds. The arterial pressure waveform consists of multiple forward and backward propagating waves. A distortion can be observed as the waveform continuously changes during the movement of blood along the arterial tree. Due to changes in arterial characteristics or vessel architecture, the phenomenon of reflection occurs in many areas of the arterial tree. As the pressure waveform moves through large elastic arteries and smaller conductive arteries, pulse pressure is amplified due to the decreasing compliance of the conductive vessels and wave reflection.

Pulse pressure amplification is actually a distortion rather than an amplification, since it does not require additional energy input into the arterial system. Therefore, the peripheral pulse pressure tends to overestimate central pulse pressure. The pulse pressure waveform decreases as it reaches smaller arteries, arterioles, and capillaries. This peripheral reduction is called damping, and is primarily determined by changes in vascular resistance and the compliance of these smaller vessels. Systolic arterial pressure (SAP) is the maximum pressure that occurs in the arterial system during ventricular systole. It is determined by factors such as left ventricular contractility, SV, and arterial elastance. Diastolic arterial pressure (DAP) is the minimum pressure measured in the aorta during the diastolic phase, which starts with the closing of the aortic valve. Although the effect of the left ventricle on the arterial system, ends with the closure of the aortic valve, blood flow and perfusion continue at a different level. In terms of coronary perfusion, this pressure perfuses both coronary arteries and the rest of the arterial system when the aortic valve closes. This pressure is influenced by factors like arterial elastance and other afterload determinants, which also determine the SV passing into the aorta during systole. Mean arterial pressure (MAP) is the average pressure in the arterial system during the systolic and diastolic phases, and it reflects the pressure required to provide tissue perfusion. It can be calculated using this pragmatic formula: MAP=DAP+1/3 (SAP-DAP). However, this formula assumes that, physiologically, each cardiac cycle consists of one unit of systolic duration and two units of diastolic duration. Of note, SV, CO, SVR, arterial elastance, and total blood volume can influence MAP. In cases of HR changes, arrhythmias, heart failure, or aortic valve pathologies, the formula-based calculation of MAP may not reflect the true value and is valid only within normal physiological limits. In these cases, arterial wave analysis and calculating the area under the curve for MAP would be more accurate approaches.

Pulse pressure is the difference between systolic and diastolic pressure (approximately 40 mmHg) and is used as an indicator of arterial stiffness. Pulse pressure is related to the elasticity of the arterial walls and the SV of the heart. A narrowed pulse pressure (below 25 mmHg) may indicate conditions like aortic stenosis, severe hypovolemia, heart failure, or tamponade. On the other hand, in of conditions such as arteriosclerosis, septic shock, aortic insufficiency, pregnancy, or hyperthyroidism, an expanded pulse pressure range above 100 mmHg may be observed.^6^

The venous system, which holds approximately 70% of the total blood volume, exhibits a compliance 30 times greater than that of the arterial system, allowing it to accommodate large volumes of blood with minimal increases in pressure, thus acting as a reservoir for regulating circulatory dynamics. This makes it quite difficult to establish a behavioral model for the venous system.^7^ However, it is well known that evaluating circulatory and systemic filling pressures, particularly CVP, provides valuable information for the anaesthesiologist’s fluid management and hemodynamic manipulations. The venous system maintains a certain amount of blood volume to prevent the collapse of the vascular bed. The volume that keeps the vessel open is known as the unstressed volume. When the amount of blood in the venous system exceeds a certain level, it creates an elastic tension in the vessel wall, facilitating blood flow towards the right atrium. This volume is known as the stressed volume. The venous return to the right atrium is determined by the difference between the systemic filling pressure created by the stressed volume, which facilitates blood movement toward the right atrium, and the right atrial pressure that must be overcome. The higher the filling pressure and the lower the right atrial pressure (not falling below zero), the better the venous return becomes. Conversely, when the filling pressure is low (due to low stressed volume, hypovolemia, or vasodilation) and the right atrial pressure is high (due to heart failure), venous return is impaired. The mean systemic filling pressure (Pmsf), unlike circulatory filling pressures, is the pressure at which the arterial and venous system pressures are equalized in any part of the system other than the heart and pulmonary circulation. This pressure can be measured using the Pmsf hold or the Pmsf analog technique.^8^

### Blood flow

The volume passing through a given cross-sectional area per unit time is called flow, and a pressure gradient is required to overcome a certain amount of resistance for the flow to occur (Flow=ΔP/R). Flow characteristics are classified as “laminar”, “turbulent”, and “transitional” flows, determined by the Reynolds number. The Reynolds number is a unitless term reflecting a critical limit at which flow shifts from laminar to turbulent flow, and is influenced by the density, velocity, viscosity of the fluid, and the diameter of the vessels. When the Reynolds number is below 2,000, laminar flow is observed; between 2,000 and 4,000, transitional flow occurs; and above 4,000, turbulent flow is present.

Viscosity is the resistance created by the liquid against flow, and its unit is centipoise (cP). The viscosity of blood ranges from 2.3 to 5.6 cP. The viscosity of blood is said to be higher* in vitro* than *in vivo*, which is attributed to the greater axial linear flow tendency in vascular structures.^9^ In human physiology, systemic arterial blood flow cannot be described purely as “laminar” or “turbulent”. Considering the pumping function of the heart and the total vascular network, concepts such as arterial compliance, arterial resistance, and aortic impedance come into play. To address this concept comprehensively, the “arterial Windkessel” model was developed over the years. The term “Windkessel” is German for “air chamber” and refers to hydraulic accumulators used by fire brigades to extinguish fires. In short, large arteries, due to the elastic fibers in their tunica media, behave like reservoirs, distending during systole and continuing to supply blood flow to the periphery during diastole. This describes arterial compliance. Peripheral small arteries and arterioles that create arterial resistance are another factor influencing flow. When aortic impedance due to disturbed aortic pressure in diastole, and total arterial inertance (flow inertia) in low-frequency cycles, is added to these two concepts, a four-element Windkessel model is formed.^10^ After blood is pumped into the aorta under high pressure, it passes first through the large and medium arteries, then to smaller feeding arteries, the terminal arterioles, and the pre-capillary resistance arterioles, completing the arterial transition. Then, it enters the true capillaries, which have no contractile properties, followed by post-capillary resistance venules and collecting veins. Finally, the circulation is completed through the venous capacitant veins and large veins, which have a high capacity for volume. Large and medium-sized arteries and their subsequent arterioles are vascular structures that conform to the Windkessel model. Vascular smooth muscle cells are present in the outer layer of arteries, arterioles, and large veins. Depending on the “tone” (tension) of these cells, blood flow is “regulated” (autoregulatory). In venules and collecting veins, the regulatory capacity is quite small, but they have been found to influence flow to some extent.^10^

At the organ level, the right heart, lungs, and left heart are serially connected organs that form a mechanism through which deoxygenated blood is oxygenated. In contrast, the brain, coronary vessels, gastrointestinal system, kidneys, skeletal muscles, and skin are “parallely connected” and do not receive deoxygenated blood from other organs. These organs are subject to autoregulation.^11^

### Oxygen delivery (DO_2_)

Oxygen is transported in the blood in two ways. Approximately 98% of total oxygen is bound to hemoglobin, while 2% is dissolved directly in the plasma. An oxygen molecule binds to the iron atom of the heme group, enabling each hemoglobin molecule to carry four O_2_ molecules. The sequentially increasing binding ability of oxygen to each subunit results in a unique sigmoidal oxyhemoglobin dissociation curve. Various defects in the synthesis or structure of red blood cells, hemoglobin, or the globin polypeptide chain can impair the blood’s oxygen-carrying capacity, leading to hypoxia. Factors contributing to the rightward shift of the oxygen-hemoglobin dissociation curve, supporting the unloading of oxygen at tissues, include an increase in body temperature, hydrogen ions, and 2,3-diphosphoglycerate. DO_2_ to tissues is the oxygen supplied to tissues per minute, and it is dependent on CO and the oxygen content (CaO_2_) of arterial blood defined by formula as follows: DO_2_=COxCaO_2_. Venous blood entering the lungs has a partial pressure of oxygen (PvO_2_) of about 40 mmHg, and arterial blood exiting the lungs has a PaO_2_ of approximately 100 mmHg. Many organs have compensatory mechanisms for hypoxia. One such mechanism is the production of erythropoietin by peritubular fibroblasts in the renal cortex during chronic hypoxia. However, during the acute phase, the primary compensation mechanism is an increased extraction rate. The amount of oxygen delivered in the blood includes the partial pressure of dissolved oxygen, hemoglobin oxygen saturation, and the oxygen carrying capacity of hemoglobin. Henry’s law refers to the effect of ambient pressure on dissolved oxygen, stating that the amount of dissolved oxygen in plasma equals the PaO_2_ multiplied by the oxygen solubility constant in blood (0.003 mL mmHg O_2_ dL). Hyperbaric oxygen therapy increases this solubility constant (0.3 mL mmHg O_2_ dL under 3 atm pressure) making the amount of dissolved oxygen “significant” when compared to a very “neglectable” previous constant. The oxygen-carrying capacity of hemoglobin has been empirically determined as 1.34 mL O_2_ g Hb. Referred to as the Hufner constant, this capacity can be altered by physicochemical properties of the environment. The basic knowledge of “hemoglobine tends to bind more oxygen in oxygen-rich ambient” or “hemoglobine tends to release more oxygen in hydrogen-rich ambient” (defined by Bohr’s effect) are the major determinants, and therefore, oxygen carried by hemoglobine in the arterial system is expected to be around 1.39 mL O_2_ g Hb, whereas it is around 1.31 mL O_2_ g Hb in the venous system. The degree of saturation of this capacity is indicated as SaO_2_. Therefore, the total oxygen content of arterial blood is given by the formula: CaO_2_ = (1.39xHbxSaO_2_)+(0.0031xPaO_2_), with a normal value of approximately 20 mL O_2_ dL. The mixed venous oxygen content (CvO_2_) is given by the formula CvO_2_=(1.31×Hb×SvO_2_+(0.0031×PvO_2_), with a normal value of approximately 15 mL O_2_ dL.^12^

DO_2_ is the rate at which oxygen is transported from the lungs to the microcirculation, calculated as DO_2_ (mL min^-1^)=COxCaO_2_, with normal DO_2_ being approximately 1000 mL/min. If the CI is used, normal DO_2_ is approximately 500 mL min^-1^ m².

Oxygen consumption (VO_2_) is the rate at which oxygen is removed from the blood for use by tissues. Direct measurement of VO_2_ is performed through respirometry. In a resting person, normal VO_2_ is approximately 250 mL O_2_ min. VO_2_ can be calculated by rearranging the fick equation: VO_2_ (mL O_2_ min)=COx(CaO_2_-CvO_2_).

**Figure 3:**
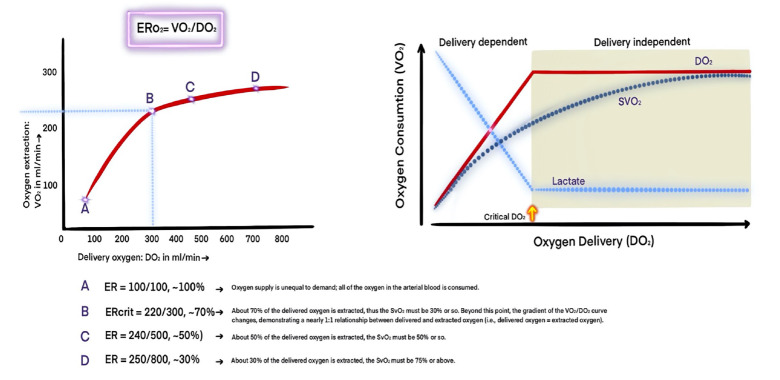
Relationship between oxygen delivery and consumption

Oxygen extraction ratio (ER) is the slope of the relationship between DO_2_ and VO_2_. The O_2_ extraction ratio=VO_2_/DO_2_. To simplify, O_2_ ER=1-SvO_2 _can be used for practical calculation. The normal O_2 _ER is between 20 to 30%. At rest, VO_2_ remains constant over a wide range of DO_2_ because changes in DO_2_ are balanced by reciprocal changes in oxygen extraction. If DO_2_ falls to a level that cannot be compensated by increasing oxygen extraction, VO_2_ starts to decrease. The DO_2 _threshold below which VO_2_ decreases is called the “critical DO_2_”, at which point VO_2_ becomes dependent on delivery. When metabolic demand increases (e.g., exercise, pregnancy, shivering, fever), VO_2_ increases because more oxygen is needed to sustain aerobic cellular metabolism. If CO or arterial oxygen content decreases, DO_2_ is expected to decrease. CO may decrease due to heart disease or hypovolemia, while CaO_2_ may decrease due to anemia or hypoxia (Figure 3).^13^

### Oxygen deficiency and hypoxia

In 1920, the classic types of hypoxia were first described as “stagnant hypoxia” (decreased CO or regional blood flow), “anoxic hypoxia” (arterial hypoxemia), and “anemic hypoxia” (decreased hemoglobin).^14^ Later, “cytopathic hypoxia” (secondary to sepsis and inflammation) and “histotoxic hypoxia” (e.g., cyanide poisoning) were also identified.^15^ Under these conditions, there is cellular failure to utilize oxygen, and increasing DO_2_ has little effect on correcting hypoxia. Physiologically, only 20-30% of the oxygen delivered to tissues is used, (O_2 _ER 0.2-0.3), and under these conditions, VO_2_ is said to be “independent of delivery”, meaning that VO_2_ is maintained despite decreasing DO_2_. However, in humans, at approximately 4 mL kg^-1^ min^-1^ of critical DO_2_ (DO_2_ critical), O_2 _ER is at its maximum (O_2 _ER 0.6-0.8), and below this level of DO_2_, VO_2_ becomes “delivery-dependent”.^12, 13^ If DO_2_ continues to fall below this critical value or VO_2_ increases at a given critical DO_2_ level, tissue hypoxia occurs. Anaerobic respiration and lactate production, resulting from the imbalance between adenosine triphosphate supply and demand, lead to type A hyperlactatemia. Type B hyperlactatemia, on the other hand, is seen in conditions such as SIRS or inflammation induced by cardiopulmonary bypass.^16^ It is known that O_2_ER increases during exercise, peaking at 0.8 during maximum exercise. This occurs because, although there is an increase in DO_2_, it does not match the increase in VO_2_ required by exercise. However, in critical illness, particularly in sepsis, VO_2_ may continue to increase despite an increase in DO_2_, and the DO_2 _critical value may be higher than normal. This is referred to as “pathological DO_2_ dependence”, and O_2 _ER may not increase in proportion to VO_2_. Additionally, in conditions where tissue edema and a decrease in functional capillary density in the microcirculation occur, even though global DO_2_ increases, sufficient oxygen may not reach the cells.^17^

### Perfusion adequacy

At the microcirculatory level, ensuring adequate perfusion and tissue oxygenation and being able to maintain it are perhaps the primary goals of optimal hemodynamic patient management. The treatments are supposed to improve tissue perfusion, but simple and pragmatic tools reflecting organ-specific perfusion are lacking. Instead, global tissue perfusion is often monitored, and systemic findings are assessed by skin color, capillary refill time (CRT), urine output, arterial pH, mixed venous oxygen saturation, blood lactate, and markers of anaerobic metabolism, such as the venous-arterial carbon dioxide difference.^18^ On the other hand, several methods have been developed for assessing regional organ perfusion, including the use of cerebral oximetry, gastric mucosal CO_2_ tonometry, tissue oxygen electrodes, sublingual tonometry etc. Yet, these methods require further clinical evaluation.^19^

### Capillary refill time

CRT is defined as the time needed for the skin to return to its normal color after 10 seconds of pressure applied to the nailbed. Normally, this time should be less than 3 seconds. Although the impact of CRT normalization on patient morbidity and mortality has not been clearly demonstrated, it is used as a quick and effective method for assessing perfusion, especially in pediatric and intensive care patients.^20^

### Mottling score

The mottling appearance on the skin suggests microcirculatory insufficiency. Those patchy color changes are observed due to heterogeneous vasoconstriction in small vessels, often starting around the knees and elbows. The mottling score can easily be assessed at the bedside, and is scored in a spectrum between 0 (no mottling) to 5 (mottling extending to the inguinal folds).^21^

### Lactate

When oxygen levels fall to critical levels, pyruvate is metabolized into lactate by lactate dehydrogenase. Therefore, lactate is considered a significant marker of anaerobic metabolism. In addition to hypoxic mechanisms, there are non-hypoxic pathways that also contribute to lactate production.^16^ Factors such as systemic inflammatory response, cardiopulmonary bypass, poisoning, catecholamines, and excessive β-adrenergic stimulation in muscle cells can increase glycogenolysis and glycolysis and lead to increased lactate production due to the saturation of pyruvate dehydrogenase enzyme activity. On the other hand, liver and kidney failure, which are responsible for lactate clearance, as well as chronic alcoholism, can also lead to increased lactate levels. Hyperlactatemia is defined as a lactate level greater than 2 mmol L, and lactic acidosis occurs when lactate levels exceed 4 mmol L along with decreasing pH. Lactate is a “late” marker of hypoperfusion but serves three purposes: (1) it can diagnose severe sepsis (infection plus high lactate); (2) it can trigger early goal-directed therapy if ≥4 mmol L; and (3) high lactate levels can guide resuscitation efforts to reduce production and enhance clearance.^22, 23^ The literature clearly suggests that high lactate levels should raise concern and serial lactate monitoring should be performed to observe lactate clearance, which may be considered as a resuscitation goal for critically ill patients.^23^

### Venous oxygen saturation (SvO_2_, ScvO_2_)

The oxygen saturation of hemoglobin in venous blood can be measured using a pulmonary artery catheter for mixed venous (SvO_2_) or a jugular central venous catheter for ScvO_2_. The normal value of SvO_2_ is between 65-80%, and a cardiac pathology causing a left-to-right shunt must be excluded before SvO_2_ measurements can be used. When oxygen extraction increases, venous oxygen saturation begins to decrease. Similarly, as oxygen extraction decreases (such as anaesthesia, increased FiO_2_, hypothermia, etc.), ScvO_2_ rises. It is a global parameter, and under physiological conditions, ScvO_2 _is typically 2-7% lower than SvO_2_. In pathological conditions, circulation centralizes to maintain brain perfusion, and SvO_2_ becomes lower than ScvO_2_. However, because of the similarity in trend graphicgraphics, the less invasiveness, and its ability to reflect the DO_2_-consumption balance, ScvO_2_ is frequently used instead of SvO_2_.^24, 25^ Yet, the reliability of ScvO_2_ for predicting SvO_2_ in severe sepsis is still a matter of debate.^26^ From another perspective, lactate can predict ScvO_2_, but only at certain critical levels in a few shock patients, it is emphasized that lactate and ScvO_2_ are not interchangeable markers of tissue oxygenation/perfusion.^27^ These findings underscore the need for comprehensive patient monitoring through the use of multiple devices, employing all available modalities to ensure optimal care.

### Near-infrared spectroscopic oximeter (NIRS)

NIRS devices are based on the modified Beer-Lambert law, which states that the intensity of transmitted light decreases exponentially as the concentration of a substance through which the light passes increases. Potential changes in perfusion and oxygenation in the frontal cortex are monitored, thus categorizing it as a regional parameter. Unlike pulse oximeters, it works even in non-pulsatile conditions. This allows continuous, non-invasive, real-time, and reliable oxygen saturation measurement even during cardiopulmonary bypass and arrest situations. Its ability to measure non-invasively is a significant advantage over jugular venous oxygen saturation measurements. Tissue oxygenation measured by NIRS is a combination of arterial, venous, and capillary blood. In frontal measurements, approximately 70-75% of the cerebral blood volume is venous.^28^ The normal range for healthy individuals is accepted as 58-82%, with measurements between 0-15% providing significant information on cardiopulmonary resuscitation processes. A decrease of 20% unilaterally or bilaterally from an individual’s baseline (before anaesthesia induction) or a 50% absolute decrease in rScO_2_ is considered pathological.^29^ In heart, and carotid surgery, unilateral decreases or increases can indicate issues like cannula malposition, shunt requirements, major stroke, or hyperperfusion. Although existing research suggests that a specific intervention or factor reduces postoperative cognitive dysfunction, more studies are needed.^30^

There are multiple potential measurement areas for peripheral tissue oxygenation with NIRS, and these are typically defined under the assumption that the underlying muscle body serves as a relatively homogeneous tissue compartment. The thenar eminence, which has a thin fat layer and is less prone to systemic edema than other skin areas, is commonly considered an ideal area for measurement.^31^ Peripheral muscle tissue oxygen saturation (StO_2_), which is also determined by NIRS, has been suggested as a more reliable indicator of traumatic shock than systemic hemodynamic or invasive oxygenation variables.^32^ The main disadvantage of the thenar eminence is its cone-like structure, which causes problems with attaching the probe. Physiologically, the forearm, rather than just the thenar, is the dominant area for circulatory distress (reflex) vasoconstriction. Vascular response in this area may occur earlier and more intensely than in other body regions. This makes the forearm a suitable area for peripheral NIRS measurement, which has been validated in the literature, detecting circulatory distress in experimental hypovolemia more sensitively than the thenar eminence.^33^ Other potential areas include the pectoral and deltoid muscles, the paravertebral region, the vastus lateralis muscle, kidneys, or intestines in neonates and infants, where NIRS monitoring can be performed. Yet, these promising indicators are not standard practice.

### Pv-aCO_2_ difference (ΔPCO_2_ gap)

The Pv-aCO_2_ gap is the difference between the partial CO_2_ pressure in venous and arterial blood, whose normal value is 2-6 mmHg. The Pv-aCO_2_ difference is proportional to CO_2_ production and inversely proportional to CO, and it shows similar trends to SvO_2_. It reflects the venous return in the capillary bed and the adequacy of microcirculation. Therefore, this particular indicator shows the adequacy of venous blood flow in order to eliminate CO_2_ rather than hypoxia; the Pv-aCO_2_ difference indirectly reflects CO. When Pv-aCO_2_ is above 6 mmHg, CO is assumed to be inadequate.^34^ However, hypoxia (except stagnant hypoxia) does not lead to an increase in the Pv-aCO_2_ gap. This can help distinguish whether perfusion disorders are circulatory or respiratory in origin. CO_2_ changes occur faster than changes in lactate levels, making PCO_2_ a more sensitive marker for hemodynamic alterations. In many critical conditions, the Pv-aCO_2_ gap has been argued to have significant predictive value for severe postoperative complications. Additionally, persistent high Pv-aCO_2_ gaps may indicate increased mortality, but it is still unclear whether closing it improves prognosis.^35, 36, 37^

### Pv-aCO_2_/Ca-vO_2_ ratio

This ratio is derived from the respiratory quotient (RQ) and indicates how many moles of O_2_ are consumed for each mole of CO_2_ produced. In aerobic metabolism, one O_2_ molecule corresponds to one CO_2_ molecule, and the RQ value is 1. In hypoperfusion, where anaerobic metabolism dominates, both VO_2_ and VCO_2_ decrease, but this decline is asymmetrical. VCO_2_ decreases negligibly due to the contribution of anaerobic metabolism, while VO_2_ increases significantly. Pv-aCO_2_/Ca-vO_2_>1.4 indicates anaerobic metabolism. At this level, a reaction is observed earlier than the change in lactate levels.^38^ However, some studies emphasize that this ratio does not predict changes in postoperative cardiac surgery patients and may not reliably predict the onset of organ dysfunction.^39, 40^

### Sublingual capnometry

In hemodynamically unstable intensive care unit patients, sublingual PCO2 (PSLCO2) and the PSLCO2-PaCO2 gradient have been found to be better predictors of tissue hypoxia than traditional markers.^41, 42^ These suggestions have not yet gained widespread recognition in the current literature.

### Future perspective in tissue oxygenation assessment

With more advanced devices, the goal is to measure several valuable parameters with a single measurement probe that can be applied at the bedside. The combination of spectrophotometry, laser Doppler-based perfusion measurement, and side-stream dark field imaging is expected to contribute to the diagnosis, improvement, and treatment of tissue hypoxia in the future.^43^

## Conclusion

Comprehensive hemodynamic monitoring is essential for optimizing patient outcomes in anaesthesia practices. The fundamental parameters of CO, SV, BP, and DO_2_ provide vital insights into the physiological state of patients and serve as the cornerstone for detecting hemodynamic instability. Understanding the interplay between these parameters, alongside advanced markers such as ScvO_2_, lactate, and Pv-aCO_2_ difference, enables timely and effective interventions, particularly in critically ill patients with complex comorbidities.

Despite the limitations of standard monitoring tools, integrating these fundamental principles into clinical practice enhances the ability to ensure adequate tissue perfusion and mitigate risks associated with perioperative complications. With advancements in technology and monitoring algorithms, future approaches are expected to bridge the gap between global hemodynamic assessments and microcirculatory insights, offering a more precise understanding of perfusion adequacy.

## Figures and Tables

**Table 1. Hemodynamic Parameters:** 

-	**Macrohemodynamic parameters**	**Microhemodynamic parameters**
**Basic**	- Electrocardiogram - Arterial blood pressure- Heart rate- Peripheral oxygen saturation - End-tidal carbon dioxide - Body temperature	- Skin color- Capillary refill time- Mottling score- Blood lactate level- Urine output
**Advanced**	- Central venous pressure- Stroke volume- Cardiac output- dP/dt- Parameters indicating fluid responsiveness- Arterial elastance and systemic vascular resistance - Oxygen extraction ratio- Pulmonary artery pressure and pulmonary capillary wedge pressure- Ventriculo-arterial coupling	- Mixed venous oxygen saturation and central venous oxygen saturation - Near-infrared spectroscopy - P(v-a) CO_2_ gap- Pv-aCO_2_/Ca-vO_2_- Sublingual capnometry- Oxygen saturation in the veins of specific organs

dP/dt, rate of pressure change (dP) over time (dt); P(v-a) CO₂ Gap, venous-arterial carbon dioxide difference; Pv-aCO₂/Ca-vO₂, ratio of central venous carbon dioxide difference to arterial-venous oxygen content difference
